# Hepatitis E outbreak in the health district of Bocaranga-Koui, Central African Republic, 2018–2019

**DOI:** 10.1186/s12879-024-09116-3

**Published:** 2024-02-19

**Authors:** Marina Prisca de Marguerite Nombot-Yazenguet, Joël Wilfried Doté, Giscard Wilfried Koyaweda, Philippe Armand Zemingui-Bembete, Benjamin Selekon, Ulrich Vickos, Alexandre Manirakiza, Emmanuel Nakoune, Ionela Gouandjika-Vasilache, Narcisse Patrice Joseph Komas

**Affiliations:** 1 Viral Hepatitis Laboratory, Institut Pasteur de Bangui, PO Box 923, Bangui, Central African Republic; 2 Enteric Viruses and Measles Laboratory, Institut Pasteur de Bangui, PO Box 923, Bangui, Central African Republic; 3 Laboratoire National de Biologie Clinique Et de Santé Publique, PO Box 1426, Bangui, Central African Republic; 4 Arboviruses, Hemorragic Fever Viruses and Zoonosis Virus Laboratory, Institut Pasteur de Bangui, PO Box 923, Bangui, Central African Republic; 5 Department of Medicine, Infectious and Tropical Diseases Unit, Hôpital de L’Amitié Sino-Centrafricaine, Bangui, Central African Republic; 6 Epidemiological Service, Institut Pasteur de Bangui, PO Box 923, Bangui, Central African Republic

**Keywords:** Hepatitis E outbreak, Molecular characterization, Bocaranga-Koui health district, Central African Republic

## Abstract

**Background:**

Hepatitis E virus (HEV) is a major public health disease causing large outbreaks and sporadic cases of acute hepatitis. We investigated an outbreak of HEV infection that occurred in September 2018 in the health district (HD) of Bocaranga-Koui, located in the northwestern part of Central African Republic (CAR).

**Methods:**

Blood samples were collected from 352 patients aged 0–85 years suspected to be infected with yellow fever (YF), according to the World Health Organization YF case definition. The notification forms from recorded cases were used. Water consumed in the HD were also collected. Human samples found negative for anti-YF IgM were then tested by ELISA for anti-HEV IgM and IgG antibodies. Positive anti-HEV (IgM and/or IgG) samples and collected water were then subjected to molecular biology tests using a real time RT-PCR assay, followed by a nested RT-PCR assay for sequencing and phylogenetic analysis.

**Results:**

Of the 352 icterus patients included, anti-HEV IgM was found in 142 people (40.3%) and anti-HEV IgG in 175 (49.7%). Although HEV infection was detected in all age groups, there was a significant difference between the 0–10 age groups and others age groups (*P* = 0.001). Elevated levels of serum aminotransferase were observed in anti-HEV IgM-positive subjects. Phylogenetic analysis showed HEV genotype 1e in infected patients as well as in the contaminated water.

**Conclusion:**

This epidemic showed that CAR remains an HEV-endemic area. The genotype 1e strain was responsible for the HEV outbreak in Bocaranga-Koui HD. It is necessary to implement basic conditions of hygiene and sanitation to prevent further outbreaks of a HEV epidemics, to facilitate access to clean drinking water for the population, to launch intensive health education for basic hygiene measures, to sett up targeted hygiene promotion activities and, finally, to ensure that formal health care is available.

## Introduction

Hepatitis E virus (HEV) is a major public health disease causing large outbreaks and sporadic cases of acute hepatitis. It is estimated that one-third of the world’s population is living in an HEV endemic area [1, 2]. HEV is the most common cause of acute viral hepatitis, in both resource-poor and wealthy countries [3]. HEV is a spherical, non-enveloped, single-stranded RNA virus belonging to the *Hepeviridae* family and the genus *Paslahepevirus* [4]. Although more than 8 HEV genotypes have been proposed to exist [5], four major HEV genotypes with 24 subtypes have been well described in humans [6, 7]: i) genotypes 1 and 2, which exclusively infect humans through fecal–oral transmission, circulate predominantly in regions with low sanitary level such as Africa, Asia, Latin America and Middle Eastern countries; ii) genotypes 3 and 4 are of zoonotic origin, predominate in developed countries [8, 9] and are globally distributed. Usually the HEV infection is self-limiting, but occasionally causes serious disease, such as fulminant hepatitis leading to neurological sequelae, spontaneous abortions, and sometimes death [10, 11]. Several outbreaks of HEV have occurred in low-income countries, often resulting in fulminant hepatitis with a case/fatality rate between 1 and 2% in the population of young adults, and this rate can increase to 20% among pregnant women during their last trimester of pregnancy [12, 13]. HEV is therefore potentially devastating in areas with degraded security situations, where access to essential sanitation is limited.

**Fig. 1:**
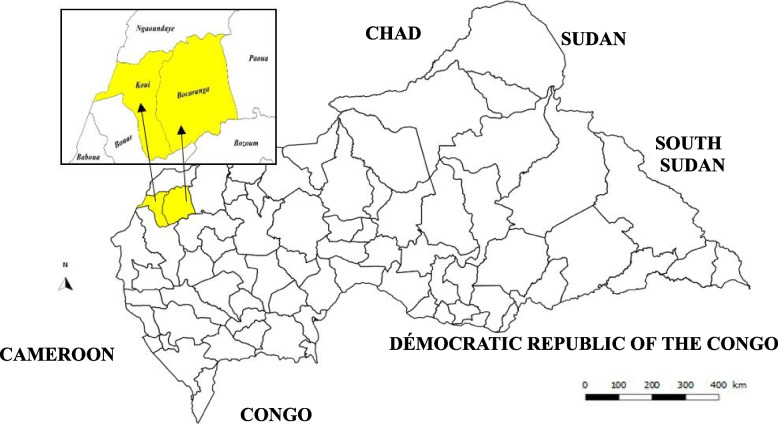
Map showing districts reporting suspect cases, northwestern Central African Republic, 2018–2019

In September 2018, an outbreak of jaundice was notified in the health district (HD) of Bocaranga-Koui, in the northwestern part of Central African Republic (CAR) (Fig. 1), through the yellow fever (YF) national surveillance system (YFNSS). In this study, we report HEV infection through differential diagnosis of YF and molecular characterization of the HEV strains identified during this outbreak.

## Materials and methods

### Studied population and data collection

Blood was collected from all individuals living in Bocaranga-Koui HD who presented with jaundice and a fever, according to the WHO YF-case definition [14]. The notification forms and blood samples were transported to the Institut Pasteur de Bangui (IPB). In addition, the clinical features observed in patients were reported by consulting physicians, and samples of water from several locations consumed by the population of these two districts were collected in sterile containers, packed with ice packs, and transported via the reverse cold chain to IPB for testing.

### Water samples preparation

Collected water samples were first processed using the 2-phase separation method with Dextran T40 and PEG 6000 and then a double treatment with 20% chloroform, according to the WHO- developed protocol for poliovirus testing for concentrating water [15]. Briefly, 500 ml of the collected water was centrifuged at 1500 g at + 4 °C for 20 min. The supernatant was collected, and the pellet was kept at + 4 °C. The supernatant (pH 7.0—7.4) was mixed with polyethylene glycol (PEG 6000), Dextran T40 and NaCl 5N. This mixture was homogenized for at least one hour and then poured into a separating funnel and kept at + 4 °C overnight. The low and intermediate phases were collected in a sterile tube. The starting pellet, together with 20% chloroform and 1 g of sterile glass ball, were added to the collected phases. The mixture was vigorously shaken and then centrifuged at 1500 g for 20 min at + 4 °C. Approximately 5 ml of supernatant was collected and used for viral RNA extraction. Extracted RNA were submitted for molecular biology tests using a real time RT-PCR assay, followed by a nested RT-PCR assay for sequencing and phylogenetic analysis.

### Biological and biochemistry testing

**Fig. 2:**
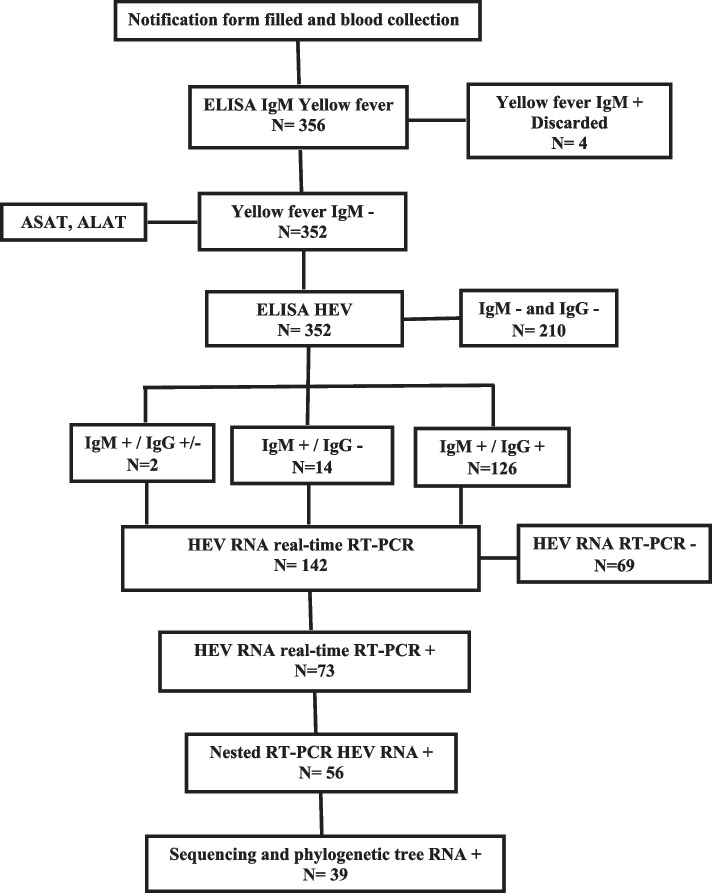
Flow chart of HEV detection in blood samples

Serum samples collected during the epidemic in the Bocaranga-Koui HD through the YF surveillance that were found negative for anti-YF IgM were retrospectively tested by HEV IgM and IgG ELISA Dia.Pro kit reference EVM.CE (Diagnostic Bioprobes srl, Milan, Italy) [16, 17] and by real-time RT-PCR, as previously described [18]. A HEV case was confirmed if the sample was positive for IgM antibodies and/or for real time RT-PCR (Fig. 2). Samples which were found positive for YF were discarded from our study (Fig. 2).

For the RT-PCR testing, viral RNA was extracted from 140 µl serum samples positive for HEV IgM ELISA and concentrated water samples, using QIAamp Viral RNA Mini Kit (QIAGEN, Courtaboeuf, France), and then retrotranscribed into cDNA using the High-Capacity cDNA Reverse Transcription Kit (Applied Biosystems, Foster City, CA, USA) according to the manufacturer's instructions. Real-time RT-PCR was carried out in 96-well plates using the TaqMan® Universal RT-PCR Master Mix Reagent (Applied Biosystems, Foster City, CA, USA), the reaction mixture contained the following ingredients: 12,5 μl of 2X PCR Master mix, 5 μl of the resulting cDNA, 1 µM for each primers and probe, and sterile water to make up the final volume of 25 µl, according to the manufacturers' recommendation. The primers and probe (10 μM) employed were: Taq HEV-F (5´-GCCCGGTCAGCCGTCTGG-3´); Taq HEV-R (5´-CTGAGAATCAACCCGGTCAC-3´); TaqHEV-S (5´-FAM- CGGTTCCGGCGGTGGTTTCT-TAMRA-3´) [18]. Real-time RT-PCR amplification was performed using an ABI PRISM® 7500 real-time PCR instrument (Applied Biosystems, Foster City, CA, USA) under the following conditions: 50 °C for 2 min and 95 °C for 10 min, followed by 45 cycles at 95 °C for 15 s and 60 °C for 1 min. A sample was considered positive if the cycle threshold (*C*_*T*_) was < 37 amplification cycles.

For all suspected cases, serum samples were analyzed for alanine aminotransferase (ALAT) and aspartate aminotransferase (ASAT) using ABX Pentra 400 (RAB1251FR).

### Phylogenetic analysis

A nested RT-PCR amplifying a 348-bp portion of the open reading frame 2 region was performed as previously described [19] on RNA samples that tested positive with the real-time RT-PCR using the thermocycler Gene Amp PCR System 97,000 (Applied Biosystems). Briefly, 2 mM of each primers 3156N (5’-AATTAGCYCAGTAYCGRGTTG-3’) and 3157N (5-CCCTTRTCYTGCTGMGCATTCTC-3’) and Titan One Tube RT-PCR kit (Roche, diagnosis, Germany) were used. Cycling for the first reaction were as follows: 50 °C for 30 min, 94 °C for 2 min followed by 40 cycles of 94 °C for 30 s, 42 °C for 1 min, 68 °C for 1 min, and a final extension at 68 °C for 7 min. The second reaction was performed with 01 mM of each primers 3158N (5’GTWATGCTYATWCATGGCT-3’) and 3159N (5’- AGCCGAAATCAATTCTGTC-3’) and Taq DNA polymerase Kit (Roche, Diagnosis, Germany) were used. Cycling for the second reaction were as follows: 94 °C for 3 min, 40 cycles at 94 °C for 45 s, 42 °C for 45 s, and 72 °C for 45 s, and a final elongation step at 72 °C for 5 min. Amplified PCR products of the second reaction were separated by 3% (w/v) agarose gel electrophoresis. The amplicons were purified using QIAquick PCR Purification Kit (QIAGEN, Hilden, Germany) and then sent to GATC Biotech (Konstanz, Germany) for direct sequencing. Phylogenetic analysis of the partial ORF2 gene of HEV was conducted with MEGA7 software (www.megasoftware.net) and aligned by CLUSTAL Muscle algorithm. A Phylogenetic tree was constructed using the neighbour-joining method and the Kimura-2 model with 1000 bootstrap replicates using MEGA 7 [20]. HEV reference strains of genotypes/subtypes were included [21]. The sequences obtained in this study were deposited in GenBank with accession numbers MN901844 to MN901869 and MW258967 to MW258978.

### Statistical analysis

Data analysis was performed using STATA version 14 (Stata Corp LP, College Station, TX, United States). Odds ratios (OR) and their respective 95% confidence intervals (CI) were calculated for each association. Pearson chi-squared or, when necessary, Fisher exact tests were used to compare distribution for categorical variables for the different groups. Statistical significance was assumed at *P* < 0.05 in the univariate analysis.

## Results

Blood samples were collected from 352 people living in the Bocaranga-Koui HD, including 172 women (48.3%) and 180 men (51.7%), with a sex ratio of 1.07, aged between 0 and 85 years (mean, 22.6 years, SD ± 17.4).

The serology results reported in Table 1 shows that, by ELISA analysis, 142 people (40.3%) had HEV-positive IgM antibodies, indicating that a high proportion of the population had an ongoing HEV infection, with more infected women (43.3%) than men (38.9%). A similar observation was made in the case of HEV IgG antibodies, with 53.0% of women infected and 50.6% of men. The difference observed between genders was not statistically significant in either case (*P* = 0.39). However, the distribution of HEV serology (IgM and IgG) showed a statistically significant difference according to age group, with the 21–30-year-old age group and those over 40 years of age being significantly more positive for HEV IgM antibodies than the 0–20 year-old and 31–40 year-old groups (*P* = 0.001).

**Table 1:** Demographic Characteristics and serum aminotransferase levels for HEV infection in 352 people with acute jaundice

	IgM anti-HEV				IgG anti-HEV			
Characteristics	Positive, *No*. (%)	Negative, *No*. (%)	OR (95% CI)	*P*	Positive, *No*. (%)	Negative, *No*. (%)	OR (95% CI)	*P*
Prevalence	142 (40.3)	204 (57.9)			175 (49.7)	163 (46.3)		
Sex				0.39				0.65
Male	70 (38.9)	110 (61.1)			87 (50.6)	85 (49.4)		
Female	72 (43.3)	94 (56.7)			88 (53.0)	78 (47.0)		
Age group				0.001				0.001
0–10	16 (14.9)	91 (85.1)	-		18 (17.9)	83 (82.1)	-	
11–20	27 (42.1)	37 (57.9)	4.1 (2.0–8.6)	0.001	37 (56.0)	29 (44.0)	5.8 (2.9–11.8)	0.001
21–30	42 (59.1)	29 (40.9)	8.3 (4.0–16.9)	0.001	51 (71.9)	20 (28.1)	11.7 (5.6–24.3)	0.001
31–40	25 (49.0)	26 (51.0)	5.5 (2.5–11.8)	0.001	31 (63.2)	18 (36.8)	7.9 (3.6–17.1)	0.001
> 40	32 (61.5)	20 (38.5)	9.2 (4.2–19.8)	0.001	38 (74.5)	13 (25.5)	13.4 (5.9–30.3)	0.001
AST^a^, IU/L				0.001				0.19
< 15	2 (18.1)	9 (67.0)	0.5(0.1–2.5)	0.40	2(22.2)	7(77.7)		0.90
15–46	28 (30.4)	64(72.4)	-		49(53.8)	42(46.1)		
> 46	112 (46.0)	131(50.0)	1.9(1.1–3.2)	0.01	124(52.1)	114(47.9)		0.91
ALT^b^, IU/L				0.001				0.001
< 11	7 (25)	21 (75)	1.0 (0.4–2.7)	0.41	09(36)	16(64)	0.7(0.3–1.7)	0.49
11–66	41 (23.7)	132 (76.3)	-		73 (43.2)	96(56.8)	-	
> 66	94 (64.8)	51 (35.1)	5.9 (3.6–9.7)	0.001	93(64.5)	51(35.4)	2.3(1.5–3.7)	0.001

*Abbreviations*: *CI* Confidence Interval, *HEV* hepatitis E virus, *OR* odds ratio

^a^AST, aspartateaminotransferase, Normal range: 15–46 IU/L

^b^ALT, alanine aminotransferase, Normal range: 11–66 IU/L

Among those who were IgM anti-HEV antibody positive, three patients (a 30-year-old pregnant woman, a 35-year-old man and a 12 month-old child) died during this epidemic.

More than 50% of patients who were anti-HEV IgM antibody positive had higher than average ALT (59.8%) and AST (71.8%) values during this epidemic (Table 1). Dual amplification by real-time RT-PCR and nested RT-PCR resulted in 73 (51.4%) of the 142 sera being amplified as IgM anti-HEV positive (Fig. 2).

The clinical characteristics and source of drinking water of patients who were anti-HEV IgM positive during this epidemic are described in Table 2.

**Table 2:** Clinical features and additional parameters of study population

Characteristics	Anti-HEV IgM
	**Positive (*n* = 142)**
**Signs and symptoms**	
Jaundice (*n* = 352)	102 (71.8%)
Fever (*n* = 276)	89 (62.6%)
General fatigue (*n* = 98)	17 (11.9%)
Abdominal pain (*n* = 82)	11 (7.7%)
Loss of appetite (*n* = 102)	09 (6.3%)
Dark- colored urine (*n* = 18)	11 (7.7%)
**Additional parameters**	
**Drinking water source**	
Well (*n* = 218)	97 (68.3%)
Handpump (*n* = 51)	6 (4.2%)
Handpump/well (*n* = 23)	12 (8.4%)
Municipal tap (*n* = 32)	10 (7.0%)
Others^a^ (*n* = 28)	15 (10.5%)

^a^Drilling/pump/well/ backwater

**Fig. 3:**
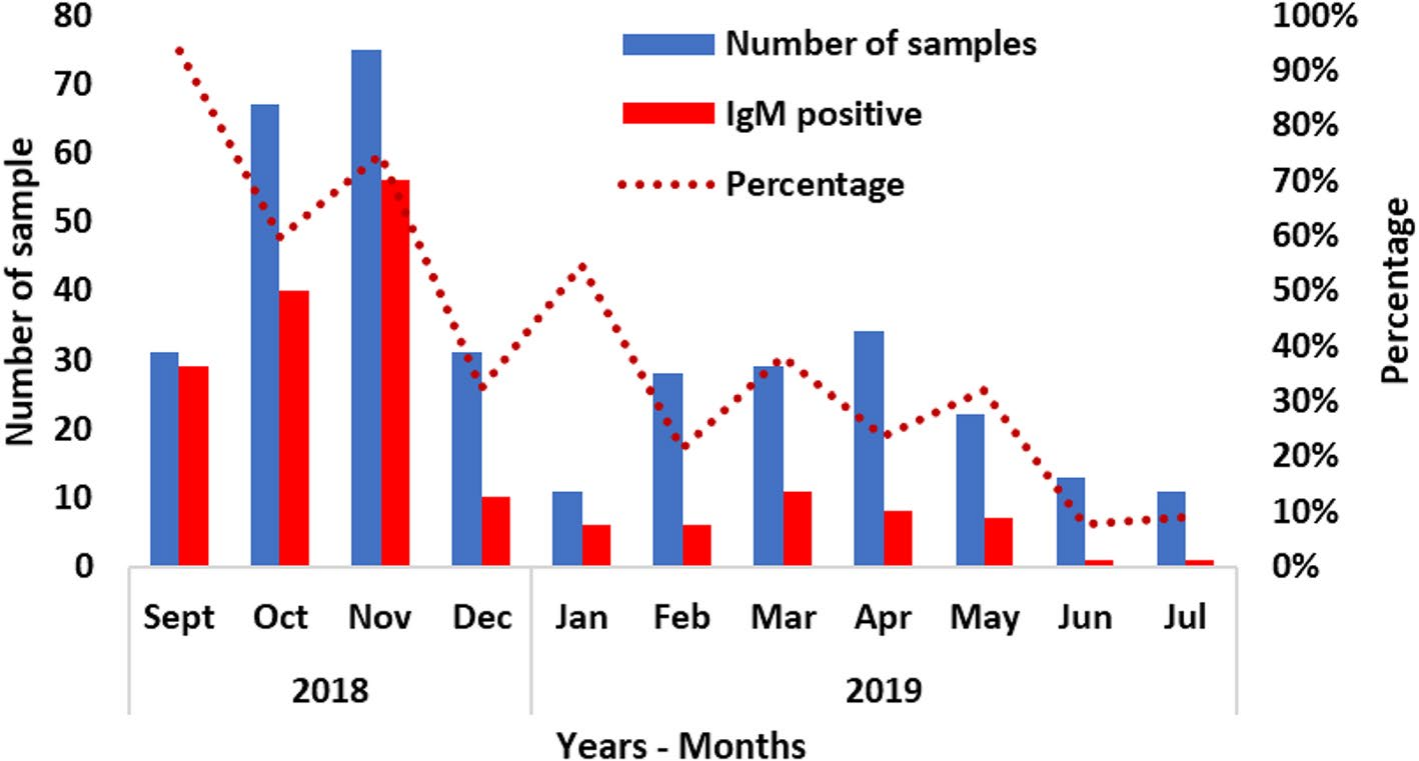
Distribution of anti-HEV IgM per month (From September 2018 to July 2019)

The clinical symptoms most frequently observed in these patients were jaundice (71.8%), followed by fever (62.6%), and other signs such as general fatigue (11.9%), abdominal pain (7.7%), dark-colored urine (7.7%), and loss of appetite (6.3%). Regarding the origin of water drunk by anti-HEV IgM antibody-positive patients, well water was the most frequently consumed (68.3%), followed by other water sources (10.5%), and the combination of well water and hand-pump water (8.4%). It should be noted that at least 7% of people who consumed only public fountain water were also found to be IgM antibody positive, and only 4% of these patients consumed only pump water. Determination of the persistence of infection in the population was measured from September 2018, considering the variation in positive anti-HEV antibodies IgM (Fig. 3). The highest prevalence was recorded in November, three months after the onset of the epidemic. From December 2018, prevalence fell back to a lower level and remained constant until May 2019.

### HEV genotyping

**Fig. 4:**
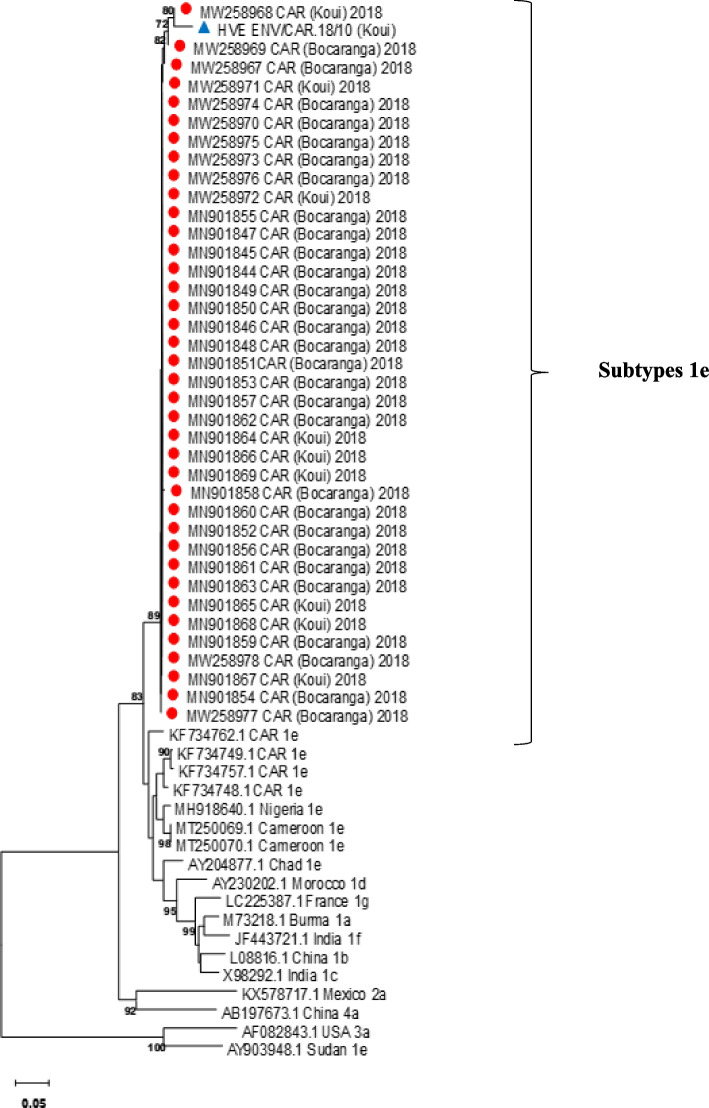
Phylogenetic analysis of sequences obtained in the study in comparison to reference sequences from GenbanK. Strains isolated from the Bocaranga and Koui patients samples during the 2018 epidemic are indicated by a red circle  and from the water well sample by a blue triangle  . HEV genotype 1e sequences obtained in the present study were deposited in the NCBI GenBank database on the accession numbers MN901844 to MN901869 and MW258967 to MW258978. Bootstrap values are indicated when > 70% as a percentage obtained from 1000 re-samplings of the data

In total, 39 out of the 73 samples that were positive by RT-PCR and one water sample from one of the wells out of the 52 water samples used by the patients were amplified and sequenced. Phylogenetic analysis of the sequences (Fig. 4) showed that the HEV strains isolated from the different samples belonged to genotype 1e and were close to strains already isolated in 2008 and 2009 during previous epidemics in CAR [22].

## Discussion

During the hepatitis E epidemic in the HD of Bocaranga-Koui, over 40% of sampled people were recently infected (IgM) with HEV, and 49% had already been in contact with this virus. This prevalence in patients with acute jaundice shows that hepatitis E is endemic in the CAR causing major epidemics [22–24].

This high prevalence is not surprising, because the Bocaranga-Koui HD is in an area of social instability, with persistent military-political conflicts that have long-term effects. In addition, the region has a very high crime rate, forcing most of the population to live in the bush or in host communities where hygiene conditions are precarious. Among those infected, more women than men carried HEV antibodies, although we found that this difference was not statistically significant. This observation has been reported in previous studies [25], in which more women than men were infected during these epidemics. In our study, this high prevalence of HEV infection among women can be explained by the fact that most household activities are carried out by women in this rebel-held health district. In addition, to avoid capture by rebels, many men have left this area for security reasons. In any case, this observation confirms that HEV infection is not inherently linked to gender, but rather because of it is waterborne and thus linked to certain professional and/or household activities [25]. The proportion of HEV cases during this epidemic varied with age, but was not significantly different. Two age groups, namely those aged 21 to 30 years old and those over 40 years old were more infected than those under 21 years old and between 30 and 40 years old. This finding may result from the fact that people 21–30 and over 40 are more active in daily activities of caring for their families and may therefore be in more frequent contact with this virus, especially during epidemics. This situation may be aggravated by insecurity in densely populated sites, where hygiene conditions are far from optimal, as previously reported [26]. Data from the UN Office for the Coordination of Humanitarian Affairs (OCHA) estimates that 146,251 people lived in this health district in 2018, and that over 25,000 are "internally displaced persons, IDPs" living in conditions of humanitarian distress. In addition, to this population are voluntary returnees from Cameroon and Chad who had taken refuge in these countries during the 2013 military-political crisis in CAR. These displaced people live under extremely precarious conditions, characterized by a lack of sanitation and hygiene services, which may account for the spread of infections from one region to another and can lead to epidemics if the population’s collective immunity is low [27].

The main risk factor for HEV contamination in this district would be drinking water. Indeed, the main sources of water consumed and used by households in this health district were untreated well water, stored rainwater, water from river springs, or the rivers themselves. The proximity of latrines to these main water sources meant that the likelihood of contamination from these sources is high, given overcrowding in the area. Indeed, the exposure to diseases among populations living in displaced persons' camps where hygiene and sanitation services are lacking has been well described in the literature [28].

Two main symptoms were reported during this epidemic, namely jaundice (71.8%) and fever (62.6%). These symptoms were similar to those reported in other HEV epidemics [9], confirming that jaundice and fever are the main symptoms of this infection in HEV-endemic areas. It is therefore important to undertake a differential diagnosis between yellow fever and HEV infection whenever these symptoms are present, so that the infection can be managed properly at an early stage.

Transaminase levels were two to five times higher than normal in HEV-infected individuals, confirming the presence of hepatic cytolysis that usually accompanies HEV infection [29].

This epidemic resulted in three deaths during the time frame of the study, giving a case-fatality rate of 0.8%. Among these deaths was a woman in her last trimester of amenorrhea who died following a spontaneous abortion, probably due to HEV infection as previously reported [30]. This is not surprising, as pregnant women constitute a high-risk group, with a mortality rate in the third trimester of pregnancy of around 20% [12]. It is likely that there were other cases of death linked to this epidemic that were not identified in this study.

The epidemic curve, based on the presence of detected HEV antibodies, enabled us to assess the intensity and duration of the epidemic. Many more people had been infected with HEV in the first three months of the epidemic, between September to December 2018, coinciding with the rainy season as reported in previous studies [22].

Molecular analysis of HEV strains, obtained from infected patients, revealed that genotype 1e was the source of contamination during this epidemic. These results from our phylogenetic analysis are consistent with previous findings regarding the subtypes circulating in the CAR [22, 31] and neighboring countries [32, 33], and with waterborne transmission usually associated with HEV genotypes 1 and 2. Our genotypes 1e strains formed a cluster which were close to those found in the country during the 2008–2009 outbreak and in neighboring countries [22, 32, 34]. Our results show that HEV evolves very little and has circulated endemically in this area for almost 15 years. This demonstrates the capacity of HEV to persist for many years in the environment and because transmission is essentially fecal–oral, to cause an epidemic under certain conditions, notably low levels of hygiene, political and social insecurity and the contamination of the water supply.

We investigated the origin of the infection by analyzing water from HD wells. The results of the molecular analysis revealed only one positive water sample from one of the wells. The most likely wells water contaminated with human, or animal feces could be a major source of the epidemic, especially because wells are the main sources of drinking water. Although we found HEV in water samples from just one well, it is likely that HEV transmission during this epidemic may have resulted from well water consumption and then amplified by direct human-to-human transmission, as previously demonstrated [35]. Fecal contamination of tap water as the source of the hepatitis E epidemic has been reported in various studies in regions where HEV is endemic [22, 24, 32, 36]. The cessation of the epidemic after the distribution of non-food item (NFI) kits, the treatment of water points in the district, and awareness-raising on good hygiene and sanitation practices all support the hypothesis that fecal contamination of water was the source of this epidemic.

This epidemic showed that CAR remains an HEV-endemic area. In such regions, the eruption of an epidemic takes just a small perturbation in hygiene and sanitation conditions to trigger an HEV epidemic. The differential diagnosis of HEV/yellow fever in all cases of conjunctival icterus accompanied by fever must be carried out as a matter of urgency, to detect early cases of HEV infection, and thus to curb the onset of a hepatitis E epidemic at an early stage. However, for full operative results, it is also necessary to implement the minimum conditions of hygiene and sanitation to prevent the outbreak of a hepatitis E epidemic, to facilitate full access to clean drinking water, carry out educational campaigns on basic hygiene measures, sett up targeted hygiene promotion activities and, finally, to ensure that formal health care is available to all. All of these measures require a peaceful social and political environment.

## Data Availability

All data generated or analyzed during this study are included in this published article. The sequences obtained in this study were deposited in GenBank with accession numbers MN901844 to MN901869 and MW258967 to MW258978.

## Abbreviations

ALT: Alanine aminotransferase
AST: Aspartate aminotransferase
CAR: Central African Republic
HD: Health district
HEV: Hepatitis E virus
IgG: Immunoglobulin G
IgM: Immunoglobulin M
IPB: Institut Pasteur de Bangui
NFI: Non Food Items
OCHA: UN Office for the Coordination of Humanitarian Affairs
OR: Odds ratios
RT-PCR: Reverse transcriptase-polymerase chain reaction
WHO: World Health Organization
YF: Yellow fever
YFNSS: Yellow fever national surveillance system
